# Inflammatory Markers and Plasma Fatty Acids in Predicting WBC Level Alterations in Association With Glucose-Related Markers: A Cross-Sectional Study

**DOI:** 10.3389/fimmu.2020.00629

**Published:** 2020-04-14

**Authors:** Gurum Shin, Kyunghye Jang, Minjoo Kim, Jong Ho Lee, Hye Jin Yoo

**Affiliations:** ^1^Department of Food and Nutrition, College of Human Ecology, National Leading Research Laboratory of Clinical Nutrigenetics/Nutrigenomic, Yonsei University, Seoul, South Korea; ^2^Department of Food and Nutrition, College of Life Science and Nano Technology, Hannam University, Daejeon, South Korea; ^3^Research Center for Silver Science, Institute of Symbiotic Life-TECH, Yonsei University, Seoul, South Korea

**Keywords:** white blood cell count, plasma fatty acids, inflammatory markers, glucose-related markers, gas chromatography-mass spectrometry

## Abstract

Aging leads to immune function changes which contribute to occurrence of chronic conditions. White blood cell (WBC) level is a marker widely known to reflect the immune function, thus, prediction of WBC level changes by using certain biomarkers is needed to prevent chronic conditions and to decrease the burdens of aging. In this respect, the present study aimed to explore the relationships between inflammatory markers and plasma fatty acid (FA) composition according to WBC levels for verifying potential predictors of WBC levels. Study subjects were divided into three groups according to their WBC count: moderate-low WBC (MLW), normal WBC, and moderate-high WBC (MHW). Inflammatory markers were measured, and plasma FA profiles were constructed via gas chromatography-mass spectrometry (GC-MS). In the MHW group, insulin, homeostatic model assessment of insulin resistance (HOMA-IR), γ-glutamyltransferase (GGT), high-sensitivity C-reactive protein (hs-CRP), and interferon (IFN)-γ showed significant increases compared to those in the other groups. In addition, the granulocyte-to-lymphocyte ratio (GLR) significantly increased according to the WBC levels, whereas the platelet-to-lymphocyte ratio (PLR) showed the opposite result. Total ω-3 polyunsaturated fatty acids (PUFAs) showed significant differences among the groups. Regarding ω-6 PUFAs, dihomo-γ-linolenic acid and docosatetraenoic acid levels were significantly increased in the MHW group compared to the other groups. Finally, multivariate linear regression analysis revealed that GGT, hs-CRP, IFN-γ, ω-3 PUFAs, and dihomo-γ-linolenic acid were independent factors for altering WBC levels. In conclusion, elevated WBC levels accompanied by an increased GLR and a decreased PLR were associated with the risk of type 2 diabetes based on increased insulin and HOMA-IR levels and decreased adiponectin levels. Additionally, GGT, hs-CRP, IFN-γ, ω-3 PUFAs, and dihomo-γ-linolenic acid levels emerged as independent biomarkers for predicting WBC level alterations. Therefore, this study showed that these inflammatory markers and plasma FAs not only affect WBC level alterations but also may play roles in the risk of type 2 diabetes as one of the chronic conditions by certain mechanisms, which should be further studied. Finally, checking these biomarkers along with WBC levels can be helpful to prevent the chronic conditions.

## Introduction

Aging is closely connected to chronic conditions, which include both chronic diseases and impairments (1, 2); chronic conditions are possibly related to aging-induced alterations of immune functions associated with inflammation in the body. Becoming an aging society, burdens such as the prevalence rate and mortality of most cancers, type 2 diabetes, and cardiovascular diseases are increasing rapidly (1). Therefore, it is important to prediagnose and prevent chronic conditions to decrease the burdens of aging (1).

Immune function can be simply assessed by white blood cell (WBC) levels, which can be changed by aging. Indeed, studies have demonstrated an association between WBC levels and aging and revealed that changes in WBC levels reflect an inflammation state in the body (3, 4). Inflammation has been considered a risk factor for chronic conditions (5). Several studies have measured inflammatory markers related to chronic conditions. Akbaraly et al. (5) found that maintaining a low interleukin (IL)-6 level may promote healthy aging by reducing the possibility of impaired respiratory and musculoskeletal function and by preventing diabetes. Reinehr et al. (6) also showed that adolescents with type 2 diabetes showed higher concentrations of inflammatory markers, such as tumor necrosis factor (TNF)-α and C-reactive protein (CRP), than those without type 2 diabetes. Furthermore, a study found that interferon (IFN)-γ shows inflammatory effects by altering the pathway of intracellular cholesterol trafficking in macrophages derived from foam cells and results in the advancement of atherosclerotic lesions (7).

In addition to inflammatory markers, studies have also revealed an association between chronic conditions and circulatory fatty acids (FAs), metabolites that very closely reflect phenotypes and metabolic changes even before phenotypic expression (8). A study on the patterns of serum FAs and desaturase using a principal component model showed that high levels of serum docosahexaenoic acid and Δ5-desaturase, as well as low levels of palmitic acid, palmitoleic acid, and Δ6-desaturase, were associated with a low prevalence of hypertension in the Chinese population (9). Therefore, inflammatory marker levels related to a chronic condition may also be associated with circulatory FA levels.

Indeed, evidence has supported that FA profiles can be related to inflammatory markers because FA levels depend on metabolic, hormonal, and/or nutritional states in individuals (10). A previous study showed that polyunsaturated FA (PUFA) profiles can reflect biochemical conditions in the human body based on their relationship with inflammatory markers (11). In addition, another study reviewed the roles of FAs in gene expression, revealing that PUFAs affect several genes involved in inflammation; i.e., the mRNA transcription of IL-1β is increased by arachidonic acid (ω-6 PUFA) and decreased by eicosapentaenoic acid and docosahexaenoic acid (ω-3 PUFAs) (12).

Based on the relationships described above, prediagnosis and prevention of chronic conditions can be strengthened by measuring not only inflammatory markers but also circulatory FAs. In this regard, studies have shown the relationships between inflammatory markers and FA profiles or between chronic diseases and FA profiles; however, most of these studies were limited to PUFAs and compared healthy individuals and patients who already had a chronic disease (10, 13, 14). Therefore, based on the findings of previous studies, the present study aimed to explore the relationships between inflammatory markers and plasma FA composition according to WBC levels in a population without any chronic diseases and to discover independent biomarkers for predicting WBC alterations, which are related to chronic condition risks. Through this study, an association among WBC level changes, inflammatory markers, and plasma FAs is finally revealed.

## Materials and Methods

### Study Population

A total of 982 individuals were recruited from the Clinical Nutrigenetics/Nutrigenomics Laboratory, Yonsei University, Seoul, Republic of Korea. The purpose of this study was carefully explained to all individuals, and written informed consent was obtained prior to their participation. The Institutional Review Board of Yonsei University approved the study protocol, which complied with the Declaration of Helsinki.

**Figure 1:**
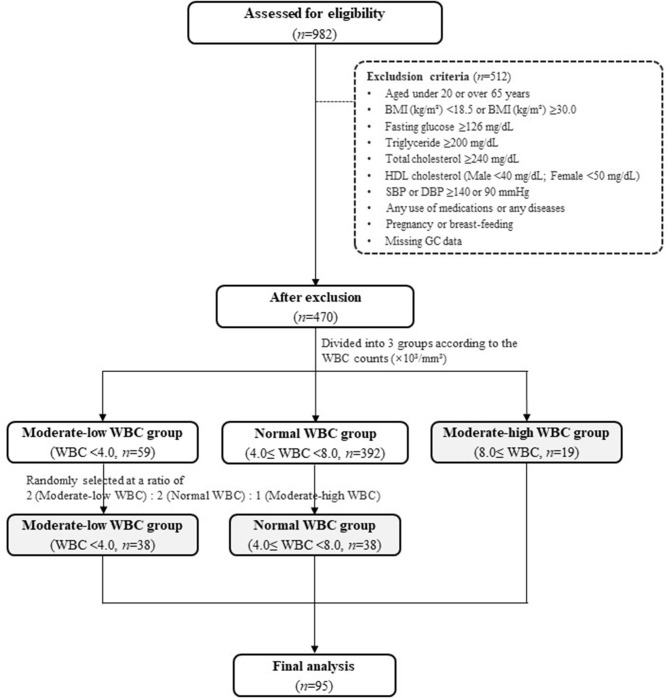
Flow chart. BMI, body mass index; DBP, diastolic blood pressure; HDL, high-density lipoprotein; SBP, systolic blood pressure; WBC, white blood cell.

Details of the eligibility assessment for study subject selection are presented in Figure 1. The study exclusion criteria were as follows: individuals aged under 20 or over 65 years; hypertension [systolic blood pressure (SBP) ≥140 mmHg or diastolic BP (DBP) ≥90 mmHg], type 2 diabetes (fasting glucose ≥126 mg/dL), dyslipidemia, cardiovascular diseases, or thyroid diseases; history of Cushing's syndrome, malignancy, or liver disease, including chronic viral hepatitis, autoimmune hepatitis, primary biliary cirrhosis, and drug-induced liver diseases; pregnant or breastfeeding; and use of any medications.

After excluding the individuals who met the exclusion criteria, 470 individuals remained. The individuals were divided into three groups according to their WBC count: a moderate-low WBC group (MLW group, WBC count < 4.0 × 10^3^/mm^3^, *n* = 59), a normal WBC group (4.0 × 10^3^/mm^3^ ≤ WBC count <8.0 × 10^3^/mm^3^, *n* = 392), and a moderate-high WBC group (MHW group, WBC count ≥ 8.0 × 10^3^/mm^3^, *n* = 19). The normal range of WBC was referenced in the previous literatures (15–18). Because the number of subjects in the MHW group was small (*n* = 19), individuals in the MLW and normal WBC groups were randomly selected at a ratio of 2:2:1 [MLW group (*n* = 38): normal WBC group (*n* = 38): MHW group (*n* = 19)] for a reliable statistical analysis. Finally, a total of 95 individuals were included as study subjects in the present study (Figure 1).

### Blood Sample Collection and Preparation

Blood samples were collected from subjects who fasted overnight before the visit. Venous whole blood specimens were obtained in EDTA-treated tubes and plain serum tubes and then centrifuged to separate serum and plasma. After separation, all of the serum and plasma aliquots were stored at −80°C prior to further analysis.

### Anthropometric and Biochemical Parameters

Weight and height were measured to calculate body mass index (BMI, kg/m^2^), and the waist-to-hip ratio (WHR) was calculated by measuring waist and hip circumference (cm). The waist circumference was measured horizontally between the lower part of the rib and the upper part of the iliac crest, and the hip circumference was measured horizontally from the widest part of the hips. After the subjects had sufficiently rested for at least 5 min, SBP and DBP were measured twice using the automatic BP monitor EASY X 800 (Jawon Medical Co., Ltd., Gyeongsan-si, Republic of Korea); the machine was stabilized over 5 min between the measurements. Average values were calculated and used for the study.

WBC (monocytes, lymphocytes, and granulocytes), red blood cell (RBC), hemoglobin, hematocrit, and platelet counts were measured using a HORIBA ABX diagnostic analyzer (HORIBA ABX SAS, Parc Euromédecine, Montpellier, France); the monocyte-to-lymphocyte ratio (MLR), granulocyte-to-lymphocyte ratio [GLR; inferred as the neutrophil-to-lymphocyte ratio (NLR)], platelet-to-lymphocyte ratio (PRL), and monocyte-to-platelet ratio (MPR) were calculated.

Levels of aspartate aminotransferase (AST), alanine aminotransferase (ALT), γ-glutamyltransferase (GGT), and serum albumin were measured by commercial enzyme-linked immunosorbent assay (ELISA) kits according to the manufacturer's instructions, and the resulting color reactions were analyzed by a Hitachi 7600 autoanalyzer (Hitachi, Tokyo, Japan).

### Glucose-Related Markers and Serum Lipid Profiles

Glucose-related markers, including fasting serum glucose and insulin, homeostatic model assessment of insulin resistance (HOMA-IR), and plasma adiponectin levels, were measured. Serum glucose levels were measured by the hexokinase method using a glucose kit (Roche, Mannheim, Germany), and serum insulin levels were measured via an immunoradiometric assay kit (DIAsource ImmunoAssays S.A., Louvain, Belgium). HOMA-IR was calculated using the following equation: HOMA-IR = [serum insulin × (fasting glucose/18)]/22.5. Plasma adiponectin levels were measured via a human adiponectin ELISA kit (Otsuka Pharmaceutical Co., Ltd., Tokushima, Japan), and the resulting color reaction was measured by a VERSA max microplate reader (Molecular Devices, Sunnyvale, CA, USA).

To confirm the subjects' lipid profiles, fasting serum triglyceride (TG), total cholesterol (TC), high-density lipoprotein (HDL) cholesterol, and low-density lipoprotein (LDL) cholesterol levels were measured. Serum TG and TC were measured via TG and CHOL kits (Roche, Mannheim, Germany), respectively, and HDL cholesterol was measured by an HDL-C plus kit (Roche, Mannheim, Germany); the color reaction was assessed with the Hitachi 7600 autoanalyzer (Hitachi, Tokyo, Japan). LDL cholesterol was calculated by the Friedewald formula: LDL cholesterol = TC – [HDL cholesterol + (TG/5)].

### Estimated Daily Energy Intake Assessments

To determine the estimated daily energy intake of the study subjects, the 24-h recall method was used. The total energy intake (kcal/d), percentage of each major nutrient (carbohydrate, protein, and fat), and estimated intake amounts of saturated FAs (SFAs, g/d), unsaturated FAs (g/d), and PUFAs (g/d) were assessed via CAN-pro 3.0 software (Korean Nutrition Society, Seoul, Republic of Korea). Total energy expenditure (kcal/d) was calculated based on physical activity data obtained from the 24-h activity records. The basal metabolic rate (BMR) was calculated using the Harris-Benedict equation.

### Inflammatory Markers

The serum high-sensitivity CRP (hs-CRP) level was analyzed with a CRP kit (Roche, Mannheim, Germany), and turbidity was measured by a Hitachi 7600 autoanalyzer (Hitachi, Tokyo, Japan). IL-1β, IL-6, and TNF-α levels were assessed via antibodies specific for each cytokine in a Bio-Plex reagent kit (Bio-Rad Laboratories, Hercules, CA, USA), and the resulting color reaction was analyzed with a Luminex 200 (Luminex Co., Austin, Texas, USA). IL-2, IL-12, and IFN-γ levels were analyzed using a human IL-2 ELISA kit (Cusabio Biotech, Houston, TX, USA), a high-sensitivity human IL-12 (p70) ELISA kit (Genway Biotech, Inc., San Diego, CA, USA), and an IFN-γ high-sensitivity human ELISA kit (Abcam plc, Cambridge, UK), respectively; the resulting color reactions were measured using a Victor × 5 2030 multilabel plate reader (PerkinElmer, Inc., Hopkinton, MA, USA) at an absorbance of 450 nm.

### Gas Chromatography-Mass Spectrometry (GC-MS) Analysis for Plasma FA Profiling

#### Sample Preparation

An internal standard was produced before the plasma sample preparation by dissolving heptadecanoic acid (Sigma-Aldrich, St. Louis, MO, USA) in n-hexane (J.T. Baker^®^ Chemicals; Avantor Performance Materials, Inc., Center Valley, PA, USA) at a 25 ppm concentration. The plasma samples (100 μL) were injected into each polytetrafluoroethylene (PTFE) screw-capped Pyrex tube; then, 500 μL of the internal standard, 2,000 μL of methanol (J.T. Baker^®^ Chemicals; Avantor Performance Materials, Inc., Center Valley, PA, USA), and 100 μL of acetyl chloride (Sigma-Aldrich, St. Louis, MO, USA) were added in sequence into the PTFE screw-capped Pyrex tubes containing the plasma samples. The mixtures were slowly vortexed and then heated at 95°C for an hour. After the heating process, the mixtures were placed on ice to cool for 10 min. After adding 5 mL of 6% potassium carbonate to the mixtures, the tubes were centrifuged (4°C, 3000 rpm, 5 min). Finally, a clear top layer (100 μL), which included the fatty acid methyl esters (FAMEs), was carefully collected and transferred into a new vial.

#### GC-MS Analysis

The prepared samples were analyzed by an Agilent Technologies 7890 N gas chromatograph coupled with an Agilent Technologies 5977 A quadrupole mass-selective spectrometer with a triple-axis detector (Agilent Technologies, Palo Alto, CA, USA). The equipment was operated in electron ionization mode at 70 eV with full-scan monitoring modes at *m/z* 50 to *m/z* 800, and the injection volume was 1 μL in the splitless mode. Derivatized samples were separated on a VF-WAX column (Agilent Technologies, Middelburg, Netherlands). Helium gas was used as a carrier in a constant flow mode (1.0 mL/min). The temperature started at 50°C (2.3 min), increased to 175°C at 50°C/min, and finally increased to 230°C at 2°C/min. To identify FAs in the samples, the relative retention times and mass spectra were compared with those of authentic standard compounds. The relative levels of the FAs (relative peak areas) were calculated by comparing their peak areas to that of the internal standard.

The FAs, which included SFAs, monounsaturated FAs (MUFAs), ω-3 PUFA, and ω-6 PUFA, and the activities of FA-related enzymes were analyzed. The enzyme activities were calculated using the following equations: Δ5-desaturase = arachidonic acid/dihomo-γ-linoienic acid; Δ6-desaturase = γ-linolenic acid/linoleic acid; C16 Δ9-desaturase = palmitoleic acid/palmitic acid; C18 Δ9-desaturase = oleic acid/stearic acid; and elongase = stearic acid/palmitic acid.

### Statistical Analysis

Statistical analysis was conducted using SPSS v.25.0 (IBM Corp., Armonk, NY, USA). For nominal variables, chi-squared tests were performed. For continuous variables, one-way ANOVA (parametric) or the Kruskal-Wallis test (nonparametric) was conducted according to the variable distribution; the method for analyzing each variable was determined by its distribution, and the nonparametric tests were performed when values did not follow a normal distribution even after a logarithmic transformation. For post hoc tests, the Bonferroni method was used. Age, sex, and weight were adjusted using ANCOVA (for variables analyzed by the parametric tests) or RANK ANCOVA (for variables analyzed by the nonparametric tests). Partial correlation coefficients, Pearson's correlation coefficients adjusted by the confounding factors (age, sex, and weight), were obtained, and a heat map was created for visualizing the correlation among the variables using MeV v.4.9.0 (http://mev.tm4.org). To verify variables that independently influenced WBC levels, a multivariate linear regression analysis with a stepwise method was performed. For every statistical analysis, two-tailed *p*-values less than 0.05 were considered statistically significant.

## Results

### Clinical and Biochemical Characteristics of the Study Subjects

As shown in Table 1, there were no significant differences in the smoking and drinking habits of the subjects. Additionally, no significant differences in most of the clinical and biochemical characteristics, including the WHR, SBP, DBP, TG, TC, HDL cholesterol, LDL cholesterol, glucose, malondialdehyde (MDA), and ALT, were observed among the three groups.

**Table 1:** Clinical and biochemical characteristics of the study subjects according to WBC levels.

	**Total (*****n*** **=** **95)**			***p^a^***	***p^b^***
	**WBC <4.0**	**4.0≤ WBC <8.0**	**WBC ≥ 8.0**		
	**(*n* = 38)**	**(*n* = 38)**	**(*n* = 19)**		
Age (year)	46.8 ± 1.72	38.0 ± 1.78	37.2 ± 3.08	0.001	-
Male/female *n* (%)	3 (7.9) / 35 (92.1)	19 (50.0) / 19 (50.0)	9 (47.4) / 10 (52.6)	<0.001	-
Cigarette smoker *n* (%)	1 (2.6)	5 (13.2)	1 (5.3)	0.198	0.599
Alcohol drinker *n* (%)	16 (42.1)	24 (63.2)	12 (63.2)	0.130	0.698
Weight (kg)	61.5 ± 1.40	69.8 ± 1.32	72.9 ± 3.24	<0.001	-
BMI (kg/m^2^)^†^	24.0 ± 0.42	25.3 ± 0.25	25.8 ± 0.64	0.016	0.767
Waist circumference (cm)	83.1 ± 1.26	87.9 ± 0.80	87.9 ± 1.99	0.006	0.097
WHR	0.86 ± 0.01	0.88 ± 0.01	0.88 ± 0.01	0.156	0.287
SBP (mmHg)	114.4 ± 1.87	117.8 ± 1.67	119.1 ± 2.96	0.243	0.971
DBP (mmHg)^†^	71.5 ± 1.37	74.3 ± 1.20	74.2 ± 2.07	0.344	0.942
TG (mg/dL)^§^	87.1 ± 5.49	91.0 ± 4.78	103.7 ± 7.88	0.170	0.474
TC (mg/dL)^§^	196.2 ± 4.37	184.7 ± 4.99	197.8 ± 5.43	0.118	0.096
HDL cholesterol (mg/dL)^§^	65.4 ± 2.23	60.0 ± 1.75	58.6 ± 2.15	0.072	0.838
LDL cholesterol (mg/dL)^§^	115.2 ± 4.34	108.8 ± 4.47	119.6 ± 5.40	0.304	0.169
Glucose (mg/dL)	85.5 ± 1.25	88.1 ± 1.16	85.4 ± 2.26	0.286	0.103
Insulin (μIU/dL)^§^	10.1 ± 0.83*^*b*^*	10.5 ± 0.58*^*b*^*	14.5 ± 1.80*^*a*^*	0.012	0.039
HOMA-IR^§^	2.14 ± 0.19*^*b*^*	2.29 ± 0.13*^*a, b*^*	3.15 ± 0.45*^*a*^*	0.019	0.049
Adiponectin (ng/mL)^§^	14.7 ± 1.88	10.1 ± 1.63	6.30 ± 0.53	<0.001	0.054
MDA (nmol/mL)^§^	7.83 ± 0.43	6.82 ± 0.32	7.01 ± 0.50	0.153	0.529
AST (IU/L)^§^	21.7 ± 0.72*^*a*^*	19.7 ± 0.81*^*b*^*	23.2 ± 1.14*^*a*^*	0.016	0.014
ALT (IU/L)^†^	16.8 ± 1.24	16.4 ± 1.11	23.6 ± 2.94	0.203	0.111
GGT (U/L)^†^	14.9 ± 0.86*^*a,b*^*	17.6 ± 1.26*^*b*^*	34.6 ± 7.75*^*a*^*	0.013	0.017

*Mean ± standard error (SE)*.

§ *variables tested following logarithmic transformation. p^a^-values of the continuous variables were derived from one-way ANOVA tests*;

† *variables tested by a nonparametric test (Kruskal-Wallis test). p^a^-values of the nominal variables were derived from the chi-squared test. p^b^-values are p^a^-values adjusted ~ for age, sex, and weight. After the adjustment, significant changes among the groups are marked with the different alphabet letters (a, b, and c) following Bonferroni post hoc test. All p-values < 0.05 were considered significant*.

*ALT, alanine aminotransferase; AST, aspartate aminotransferase; BMI, body mass index; DBP, diastolic blood pressure; GGT, γ-glutamyltransferase; HDL, high-density lipoprotein; HOMA-IR, homeostatic model assessment-insulin resistance; LDL, low-density lipoprotein; MDA, malondialdehyde; SBP, systolic blood pressure; TC, total cholesterol; TG, triglyceride; WHR, waist-to-hip ratio*.

The study subjects with high WBC counts were significantly younger than those in the other groups (*p* = 0.001), and the sex distributions among the three groups were significantly different. The weights and BMIs of the three groups increased according to their WBC count (*p* = 0.001 and *p* = 0.016, respectively); however, there were no significant differences in BMI after adjusting for age, sex, and weight (confounding factors). Likewise, the waist circumference showed significant differences among the groups before the adjustment (*p* = 0.006), but the significance disappeared after the adjustment (Table 1).

The MHW group showed a higher insulin level than the other two groups (*p* = 0.039) and higher HOMA-IR values than the MLW group (*p* = 0.049). Among the hepatic enzymes (AST, ALT, and GGT), only the AST and GGT levels were significantly increased in the MHW group after adjusting for the confounding factors (*p* = 0.014 and *p* = 0.017, respectively) (Table 1).

### Total Blood Cell Count and Inflammatory Markers in the Study Subjects According to WBC Levels

The statistical analysis results of the total blood cell counts and inflammatory markers in the three groups are shown in Table 2. With regard to the total blood cell counts, all of the parameters showed statistically meaningful outcomes. The WBC count was significantly different among the three groups. The percentage of lymphocytes in the MLW group was significantly higher than that in the normal WBC and MHW groups (*p* = 0.003). The percentage of monocytes in the normal WBC group was significantly decreased compared to those in the MLW and MHW groups (*p* = 0.001). In addition, the percentage of granulocytes in the normal WBC group was significantly higher than that in the MLW group (*p* = 0.036). The platelet count in the MHW group was significantly increased compared to those in the other groups (*p* = 0.010). Similarly, all blood cell count ratios (MLR, GLR, PLR, and MPR) also showed significant differences. The MLR in the MHW group was significantly increased compared to those in the other groups (*p* < 0.001). The GLR were not significantly different between each two groups when the Bonferroni post hoc tests were performed; however, statistical significance for all groups was observed (*p* = 0.035). A significant difference in the PLR was shown among the three groups (*p* < 0.001). Finally, the MPR in the MLW group was significantly increased compared to that in the MHW group (*p* = 0.018) (Table 2).

**Table 2:** Total blood cell counts and inflammatory markers in the study subjects according to WBC levels.

	**Total (*****n*** **=** **95)**						***p^***a***^***	***p^***b***^***
	**WBC** **<** **4.0(*****n*** **=** **38)**		**4.0** **≤** **WBC** **<** **8.0(*****n*** **=** **38)**		**WBC** **≥** **8.0(*****n*** **=** **19)**			
**TOTAL BLOOD CELL COUNTS**								
WBC (×10^3^/μL)^§^	3.49 ± 0.07^c^		5.74 ± 0.18^b^		9.08 ± 0.27^a^		<0.001	<0.001
Lymphocyte (%)^§^	41.8 ± 1.71^a^		35.8 ± 1.63^b^		31.5 ± 1.92^b^		<0.001	0.003
Monocyte (%)^§^	8.31 ± 0.76^a^		6.05 ± 0.78^b^		11.3 ± 1.80^a^		0.001	0.001
Granulocyte (%)^†^	49.9 ± 2.03^b^		58.1 ± 1.94^a^		57.2 ± 3.01^a,b^		0.003	0.036
Platelet (×10^3^/μL)^§^	227.7 ± 7.78^b^		235.4 ± 8.77^b^		268.0 ± 13.6^a^		0.028	0.010
MLR^§^	0.17 ± 0.02^b^		0.15 ± 0.02^b^		0.35 ± 0.06^a^		<0.001	<0.001
GLR^†^	1.48 ± 0.10		1.91 ± 0.13		2.09 ± 0.19		0.004	0.035
PLR^§^	174.9 ± 9.51^a^		128.0 ± 7.61^b^		100.7 ± 6.16^c^		<0.001	<0.010
MPR^§^	0.42 ± 0.02^a^		0.41 ± 0.01^a,b^		0.36 ± 0.03^b^		0.038	0.018
**INFLAMMATORY MARKERS**								
hs-CRP (mg/L)^§^	0.54 ± 0.15^b^		0.51 ± 0.06^b^		1.49 ± 0.51^a^		<0.001	0.009
IL-1β (pg/mL)	0.82 ± 0.13		0.72 ± 0.12		0.73 ± 0.12		0.835	0.867
IL-2 (pg/mL)^§^	39.1 ± 2.60		38.9 ± 2.99		54.5 ± 7.94		0.185	0.323
IL-6 (pg/mL)^†^	4.03 ± 0.62		3.45 ± 0.49		4.72 ± 1.16		0.618	0.710
IL-12 (pg/mL)^§^	61.7 ± 18.7		40.1 ± 6.05		54.9 ± 16.1		0.618	0.712
TNF-α (pg/mL)^§^	7.40 ± 0.99		9.27 ± 1.14		11.3 ± 1.57		0.091	0.149
IFN-γ (pg/mL)^§^	7.76 ± 0.97^b^		7.38 ± 0.67^a,b^		13.8 ± 3.75^a^		0.045	0.021

*Mean ± standard error (SE)*.

§ *variables tested following logarithmic transformation. p^a^-values were derived from one-way ANOVA*.

† *variables tested by a nonparametric test (Kruskal-Wallis test). p^b^-values are p^a^-values adjusted ~ for age, sex, and weight. After the adjustment, significant changes among the groups are marked the different alphabet letters (a, b, and c) following Bonferroni post hoc test. All p-values <0.05 were considered significant*.

*GLR, granulocyte-to-lymphocyte ratio; hs-CRP, high-sensitivity C-reactive protein; IFN, interferon; IL, interleukin; MLR, monocyte-to-lymphocyte ratio; MPR, monocyte-to-platelet ratio; PLR, platelet-to-lymphocyte ratio; TNF, tumor necrosis factor; WBC, white blood cell*.

Regarding the inflammatory markers, no significant differences were observed in inflammatory markers, including IL-1β, IL-2, IL-6, IL-12, and TNF-α, among the three groups. However, the levels of hs-CRP and IFN-γ in the MHW group were significantly increased compared to those in the other groups (*p* = 0.009 and *p* = 0.021, respectively) (Table 2).

### BMR, Estimated Total Energy Expenditure, and Total Energy Intake of the Study Subjects According to WBC Levels

Table 3 shows the results of the dietary patterns of the study subjects. Most of the variables, including estimated total energy expenditure, total energy intake, percentage of the major nutrients (carbohydrate, protein, and fat), and FA compositions in fat (SFAs, unsaturated FAs, and PUFAs), did not show significant differences after adjusting for the confounding factors. However, only BMR showed a significant increase in the MHW group after the adjustment (*p* = 0.034).

**Table 3:** BMR, estimated total energy expenditure, and total energy intake of the study subjects according to WBC levels.

	**Total (*****n*** **=** **95)**						***p^***a***^***	***p^***b***^***
	**WBC** **<** **4.0(*****n*** **=** **38)**		**4.0** **≤** **WBC** **<** **8.0(*****n*** **=** **38)**		**WBC** **≥** **8.0(*****n*** **=** **19)**			
BMR (kcal/d)^†^	1344.8 ± 37.4^b^		1599.1 ± 41.0^a,b^		1689.8 ± 86.4^a^		<0.001	0.034
Estimated total energy expenditure (kcal/d)^†^	1910.4 ± 46.5		2209.9 ± 50.3		2294.0 ± 94.0		<0.001	0.256
Total energy intake (kcal/d)^†^	1936.8 ± 31.8		2209.3 ± 48.3		2248.2 ± 81.8		<0.001	0.639
Carbohydrate (%)^†^	61.7 ± 0.13		61.6 ± 0.13		61.8 ± 0.16		0.799	0.823
Protein (%)^†^	15.8 ± 0.06		15.9 ± 0.06		15.8 ± 0.09		0.574	0.720
Fat (%)^†^	22.8 ± 0.18		22.7 ± 0.17		22.5 ± 0.23		0.475	0.275
SFAs (g/d)^†^	6.35 ± 0.18		7.25 ± 0.29		7.98 ± 0.44		0.001	0.271
Unsaturated FAs (g/d)^†^	10.3 ± 0.33		11.9 ± 0.46		12.7 ± 0.64		0.001	0.543
PUFAs (g/d)^†^	20.6 ± 0.74		24.9 ± 1.03		25.9 ± 1.57		<0.001	0.848

*Mean ± standard error (SE). p^a^-values were derived from one-way ANOVA*;

† *variables tested by a nonparametric test (Kruskal-Wallis test). p^b^-values are p^a^-values adjusted ~ for age, sex, and weight. After the adjustment, significant changes among the groups are marked with the different alphabet letters (a, b, and c) following Bonferroni post hoc test. All p-values < 0.05 were considered significant*.

*BMR, basal metabolic rate; PUFAs, polyunsaturated fatty acids; SFAs, saturated fatty acids*.

### Differences in Plasma FA Levels in the Study Subjects as Determined by GC-MS Analysis According to WBC Levels

Differences in plasma FA levels are shown in Table 4. Regarding SFAs, the levels of stearic acid, arachidic acid, and behenic acid were significantly higher in the MHW group than in the normal WBC group after adjusting for the confounding factors [*p* = 0.030, *p* = 0.050 (before rounding, 0.049601), and *p* = 0.040, respectively], whereas the significance of the difference in the pentadecylic acid level disappeared after the adjustment. Thus, the plasma levels of FAs that had 16 or fewer carbons were not significantly different among the three groups.

**Table 4:** Differences in plasma FA levels among the study subjects as determined by GC-MS analysis according to WBC levels.

**Relative peak area**	**Total (*****n*** **=** **95)**			***p^***a***^***	***p^***b***^***
	**WBC** **<4.0(*****n*** **=** **38)**	**4.0≤** **WBC** **<8.0(*****n*** **=** **38)**	**WBC** **≥8.0(*****n*** **=** **19)**		
**SFAs**^§^	6.361 ± 0.174	5.845 ± 0.219	6.044 ± 0.238	0.105	0.297
Lauric acid (C12:0)^†^	0.031 ± 0.004	0.023 ± 0.002	0.025 ± 0.002	0.072	0.324
Myristic acid (C14:0)^§^	0.203 ± 0.014	0.180 ± 0.011	0.219 ± 0.015	0.141	0.154
Pentadecylic acid (C15:0)^†^	0.032 ± 0.003	0.021 ± 0.003	0.029 ± 0.005	0.039	0.148
Palmitic acid (C16:0)^§^	4.220 ± 0.127	3.939 ± 0.155	3.885 ± 0.144	0.175	0.518
Stearic acid (C18:0)^§^	1.836 ± 0.047^a, b^	1.651 ± 0.057^b^	1.847 ± 0.082^a^	0.019	0.030
Arachidic acid (C20:0)^§^	0.039 ± 0.002^a, b^	0.032 ± 0.002^b^	0.039 ± 0.004^a^	0.032	0.050
Behenic acid (C22:0)^§^	0.088 ± 0.006^a, b^	0.072 ± 0.006^b^	0.092 ± 0.009^a^	0.045	0.040
**MUFAs**^§^	1.211 ± 0.050	1.123 ± 0.050	1.209 ± 0.070	0.365	0.254
Palmitoleic acid (C16:1ω7)^§^	0.141 ± 0.008	0.118 ± 0.007	0.143 ± 0.011	0.041	0.052
cis-10-Heptadecenoic acid (C17:1ω7)^§^	0.011 ± 0.001^a, b^	0.010 ± 0.001^b^	0.013 ± 0.001^a^	0.052	0.028
Oleic acid (C18:1ω9)^§^	1.049 ± 0.043	0.986 ± 0.043	1.043 ± 0.059	0.503	0.337
Eicosenoic acid (C20:1ω9)^†^	0.011 ± 0.001	0.009 ± 0.000	0.011 ± 0.001	0.112	0.179
Erucic acid (C22:1ω9)^§^	0.010 ± 0.002	0.006 ± 0.001	0.008 ± 0.001	0.126	0.096
Nervonic acid (C24:1ω9)^§^	0.045 ± 0.003	0.037 ± 0.004	0.044 ± 0.006	0.095	0.114
**ω-3 PUFAs**^†^	0.635 ± 0.042	0.402 ± 0.033	0.494 ± 0.062	<0.001	0.034
α-Linolenic acid (C18:3ω3)^§^	0.128 ± 0.011	0.087 ± 0.009	0.090 ± 0.013	0.006	0.340
Eicosapentaenoic acid (C20:5ω3)^†^	0.151 ± 0.013	0.092 ± 0.010	0.106 ± 0.018	0.002	0.601
Docosapentaenoic acid (C22:5ω3)^†^	0.036 ± 0.004	0.026 ± 0.004	0.026 ± 0.004	0.171	0.407
Docosahexaenoic acid (C22:6ω3)^†^	0.320 ± 0.025^a, b^	0.197 ± 0.018^b^	0.271 ± 0.035^a^	0.002	0.018
**ω-6 PUFAs**^†^	3.420 ± 0.168	2.842 ± 0.147	3.235 ± 0.329	0.015	0.383
Linoleic acid (C18:2ω6)^†^	2.617 ± 0.153	2.122 ± 0.121	2.460 ± 0.283	0.013	0.393
γ-Linolenic acid (C18:3ω6)^†^	0.072 ± 0.012	0.037 ± 0.008	0.055 ± 0.013	0.041	0.160
Eicosadienoic acid (C20:2ω6)^§^	0.023 ± 0.001	0.020 ± 0.001	0.024 ± 0.002	0.083	0.079
Dihomo-γ-linolenic acid (C20:3ω6)^§^	0.102 ± 0.005^b^	0.093 ± 0.004^b^	0.120 ± 0.009^a^	0.023	0.008
Arachidonic acid (C20:4ω6)^§^	0.579 ± 0.021	0.545 ± 0023	0.536 ± 0.036	0.341	0.572
Docosatetraenoic acid (C22:4ω6)^§^	0.028 ± 0.003^b^	0.025 ± 0.003^b^	0.039 ± 0.007^a^	0.059	0.005
**FA METABOLISM-RELATED ENZYMES (ACTIVITIES)**					
Δ5-desaturase^§^	6.152 ± 0.331^a^	6.031 ± 0.229^a^	4.651 ± 0.291^b^	0.002	0.001
Δ6-desaturase^§^	0.025 ± 0.004	0.016 ± 0.002	0.021 ± 0.003	0.128	0.331
C16 Δ9-desaturase^§^	0.033 ± 0.002^a, b^	0.030 ± 0.001^b^	0.036 ± 0.002^a^	0.021	0.025
C18 Δ9-desaturase^§^	0.569 ± 0.016	0.597 ± 0.015	0.564 ± 0.019	0.290	0.365
Elongase^§^	0.441 ± 0.010^b^	0.424 ± 0.007^b^	0.475 ± 0.011^a^	0.003	0.004

*Mean ± standard error (SE)*.

§ *variables tested following logarithmic transformation. p^a^-values of the continuous variables were derived from one-way ANOVA*;

† *variables tested by a nonparametric test (Kruskal-Wallis test). p^b^-values are p^a^-values adjusted ~ for age, sex, and weight. After the adjustment, significant changes among the groups are marked with the different alphabet letters (a, b, and c) following Bonferroni post hoc test. All p-values < 0.05 were considered significant. Δ5-desaturase, arachidonic acid/dihomo-γ-linoienic acid; Δ6-desaturase, γ-linolenic acid/linoleic acid; C16 Δ9-desaturase, palmitoleic acid/palmitic acid; C18 Δ9-desaturase, oleic acid/stearic acid; Elongase, stearic acid/palmitic acid*.

*FA: fatty acid. MUFAs: monounsaturated fatty acids. PUFAs: polyunsaturated fatty acids. SFAs: saturated fatty acids*.

Regarding the MUFAs, only cis-10-heptadecenoic acid exhibited a significantly higher level in the MHW group than in the normal WBC group (*p* = 0.028). The significant difference in the palmitoleic acid concentration disappeared after the adjustment; however, it still tended to be higher in the MHW group (*p* = 0.052) (Table 4).

For the ω-3 PUFAs, only the docosahexaenoic acid level in the MHW group showed a significant increase compared to the normal WBC group (*p* = 0.018). The differences in the α-linolenic acid and eicosapentaenoic acid levels lost their significance among the groups after adjusting for confounding factors. The sums of all ω-3 PUFAs were not significantly different among the three groups when the Bonferroni post hoc tests were conducted; nevertheless, statistical significance for all groups was observed (*p* = 0.034). For the ω-6 PUFAs, the levels of dihomo-γ-linolenic acid and docosatetraenoic acid were significantly increased in the MHW group compared to the other groups (*p* = 0.008 and *p* = 0.005, respectively). The significant differences in linoleic acid, γ-linolenic acid, and total ω-6 PUFA levels among the three groups disappeared after adjustment (Table 4).

Finally, with regard to the activities of FA metabolism-related enzymes, the activities of C16 Δ9-desaturase and elongase were significantly increased in the MHW group (*p* = 0.025 and *p* = 0.004, respectively). This result showed a similar tendency, as most significant plasma FAs in the MHW group were observed at higher levels than those in the other groups. However, the activity of Δ5-desaturase showed the opposite result; its activity in the MHW group was significantly lower than those in the other groups (*p* = 0.001) (Table 4).

### Relationships Among the WBC Count, Major Inflammatory Markers, and Major Plasma FAs in the Total Study Subjects

**Figure 2:**
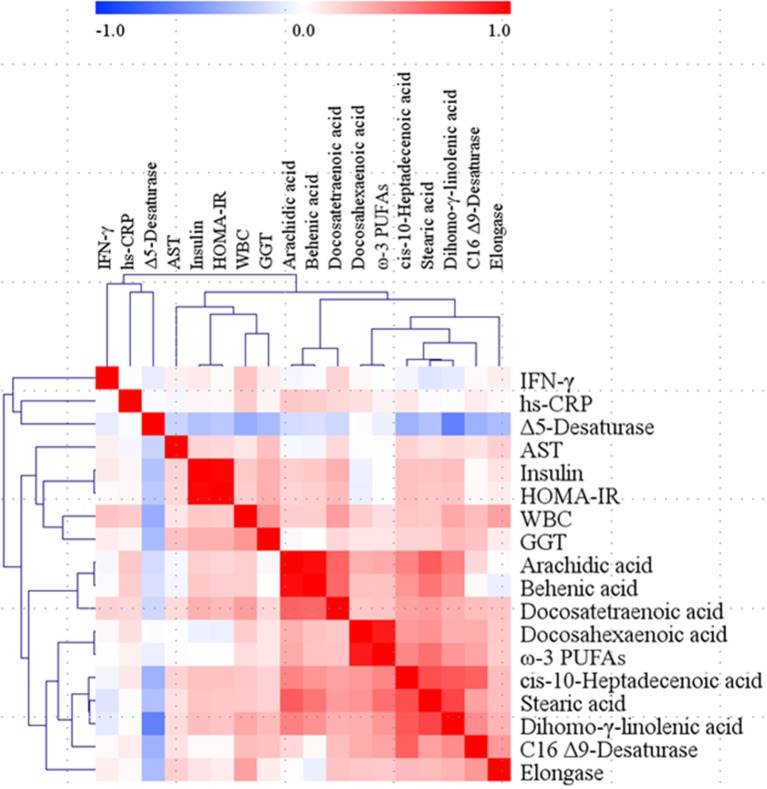
Correlation matrix among WBC, major inflammatory markers, and major fatty acids in the total study subjects. Partial correlation coefficients were obtained by adjusting age, sex, and weight for Pearson's correlation coefficients. *Red* represents a positive correlation, and *blue* represents a negative correlation. AST, aspartate aminotransferase; GGT, γ-glutamyltransferase; HOMA-IR, homeostatic model assessment of insulin resistance; hs-CRP, high-sensitivity C-reactive protein; IFN, interferon; PUFAs, polyunsaturated fatty acids; WBC, white blood cell.

Correlations among the WBC count, inflammatory markers, and plasma FAs were analyzed; some of the inflammatory markers (hs-CRP and IFN-γ) and plasma FAs (stearic acid, arachidic acid, behenic acid, cis-10-heptadecenoic acid, docosahexaenoic acid, ω-3 PUFAs, dihomo-γ-linolenic acid, docosatetraenoic acid, Δ5-desaturase, C16 Δ9-desaturase, and elongase), which showed significant differences among the groups (Tables 2, 4), were used as major variables for analyzing the correlations. In addition, insulin, HOMA-IR, AST, and GGT were used for the correlation analysis because they also showed statistically significant differences (Table 1). Pearson's correlation coefficients were adjusted by the confounding factors age, sex, and weight; thus, partial correlation coefficients were obtained. As a result, the WBC count positively correlated with the levels of insulin (*r* = 0.212, *p* = 0.044), GGT (*r* = 0.414, *p* < 0.001), hs-CRP (*r* = 0.206, *p* = 0.049), IFN-γ (*r* = 0.232, *p* = 0.026), stearic acid (*r* = 0.212, *p* = 0.042), cis-10-heptadecenoic acid (*r* = 0.224, *p* = 0.032), dihomo-γ-linolenic acid (*r* = 0.344, *p* = 0.001), docosatetraenoic acid (*r* = 0.386, *p* < 0.001), C16 Δ9-desaturase (*r* = 0.265, *p* = 0.011), and elongase (*r* = 0.371, *p* < 0.001); only Δ5-desaturase showed a negative correlation with the WBC count (*r* = −0.403, *p* < 0.001). Between each major inflammatory marker and plasma FA, only hs-CRP and arachidic acid showed a significant positive correlation (*r* = 0.219, *p* = 0.036), and tendencies for positive correlations between hs-CRP and behenic acid (*r* = 0.199, *p* = 0.057) and between IFN-γ and docosatetraenoic acid (*r* = 0.179, *p* = 0.088) were observed (Figure 2).

To identify variables that independently influenced changes in WBC levels, a multivariate linear regression analysis with a stepwise method was performed. Candidate independent variables (predictors) were the major inflammatory markers and plasma FAs mentioned above, insulin, AST, and GGT; HOMA-IR and docosahexaenoic acid were excluded because of their multicollinearity with insulin and ω-3 PUFAs, respectively. Consequently, GGT, hs-CRP, IFN-γ, ω-3 PUFAs, and dihomo-γ-linolenic acid emerged as independent factors for altering WBC levels (adjusted *R*^2^= 0.368, *p* < 0.001) (Table 5).

**Table 5:** Multivariate linear regression analysis.

**WBC**	**Total (*****n*** **=** **95)**	
	**Standardized β**	***p* [95% CI (Confidence interval)]**
GGT	0.356	<0.001 (0.025–0.072)
hs-CRP	0.203	0.023 (0.073–0.955)
IFN-γ	0.236	0.008 (0.193–1.221)
ω-3 PUFAs	−0.283	0.004 (-3.896–0.739)
Dihomo-γ-linolenic acid	0.241	0.021 (0.243–2.951)
Elongase	0.157	0.087(-0.419–6.134)

*For multivariate linear regression analysis, a stepwise method was used. Independent variable: WBC. Predictors: insulin, AST, GGT, hs-CRP, IFN-γ, stearic acid, arachidic acid, behenic acid, cis-10-heptadecenoic acid, ω-3 PUFAs, dihomo-γ-linolenic acid, docosatetraenoic acid, Δ5-desaturase, C16 Δ9-desaturase, and elongase*.

*AST, aspartate aminotransferase; GGT, γ-glutamyltransferase; hs-CRP, high-sensitivity C-reactive protein; IFN, interferon; PUFAs, polyunsaturated fatty acids; WBC, white blood cell*.

**Figure 3:**
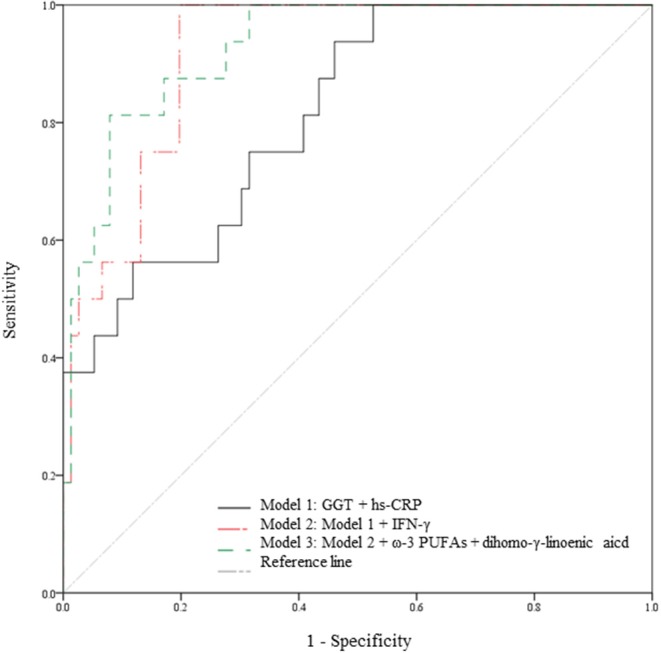
Capabilities of the variables from the multivariate linear regression analysis to predict WBC level alterations. Prediction models in the total study subjects (*n* = 95). The 5 variables (GGT, hs-CRP, IFN-γ, ω-3 PUFA, and dihomo-γ-linolenic acid) are major independent predictors for WBC count alterations; the variables were selected based on the result of the multivariate linear regression analysis. Model 1 is a prediction model consisting of conventional risk factors for WBC count alteration (81.4% *p* < 0.001). Model 2 is a prediction model with an added inflammatory marker (IFN-γ) on Model 1 (91.7% *p* < 0.001). Model 3 is a prediction model with added FAs (ω-3 PUFAs and dihomo-γ-linolenic acid) on Model 2 (92.8%, *p* < 0.001). FAs, fatty acids; GGT, γ-glutamyltransferase; hs-CRP, high-sensitivity C-reactive protein; IFN, interferon; PUFAs, polyunsaturated fatty acids; WBC, white blood cell.

The prediction models based on the multivariate linear regression analysis are shown in Figure 3. GGT, hs-CRP, IFN-γ, ω-3 PUFAs, and dihomo-γ-linolenic acid were selected as major independent predictors for WBC level alterations. Model 1 consisted of conventional factors related to WBC level alteration. Model 2 was established by adding IFN-γ, one of the inflammatory markers, to model 1. Model 3 was set by adding FAs (ω-3 PUFAs and dihomo-γ-linolenic acid) to model 2. As a result, the prediction capability was enhanced when the predictors were added [81.4% (*p* < 0.001) for model 1, 91.7% (*p* < 0.001) for model 2, and 92.8% (*p* < 0.001) for model 3].

## Discussion

Abnormal WBC levels are related to inflammation, which results in chronic conditions. Thus far, relationships between abnormal WBC levels and mortalities of chronic diseases such as cancer (19), cardiovascular diseases (20), and coronary artery disease (21) as well as between abnormal WBC levels and all-cause mortality (22) have been revealed. Indeed, Jee et al. (23) divided Korean subjects into 6 groups according to their WBC count and found that the WBC count was an independent risk factor for all-cause mortality in the Korean population. Thus, WBC levels and chronic conditions are definitely associated with each other and mediated by inflammation (24). However, the alteration of WBC levels is still ambiguous. In this regard, the present study aimed to identify potential predictors for changes in WBC levels; in particular, using GC-MS, plasma FAs were measured as metabolites (biomarkers) to assess their potential involvement in WBC level changes. Consequently, GGT, hs-CRP, IFN-γ, ω-3 PUFAs, and dihomo-γ-linolenic acid emerged as independent predictors of WBC level alterations, and WBC levels appeared to be associated with the risk of type 2 diabetes, a chronic disease.

Nakanishi et al. (4) demonstrated that a high WBC count may be a predictor of either impaired fasting glucose or type 2 diabetes, especially in nonsmokers. In addition, WBC count was reported to be associated with adiponectin (25), which is highly linked to type 2 diabetes, as it mediates insulin sensitivity (26, 27). Matsubara et al. (25) revealed that the plasma adiponectin concentration was negatively related to WBC counts in women, and the researchers also reported that decreased adiponectin concentrations could elevate HOMA-IR in nondiabetic women (28). The results of the present study showed a similar tendency to those of previous studies; WBC levels had a significant positive relationship with the insulin concentration and HOMA-IR and tended to have a negative relationship with the adiponectin concentration. In summary, elevated WBC levels may be associated with alterations of glucose-related markers such as insulin, HOMA-IR, and adiponectin; thus, individuals with high WBC levels can be considered to be at high risk for type 2 diabetes.

The difference in WBC levels in the present study was accompanied by changing ratios of each WBC component. In particular, NLR and PLR changes were studied, as they induce several chronic conditions (29–37). Lou et al. (29) showed that an increased NLR was significantly associated with IR, and the NLR values in diabetic patients were significantly higher than those in healthy controls. Likewise, Shiny et al. (38) also reported that subjects with diabetes mellitus showed significantly higher NLR values than subjects with impaired or normal glucose tolerance. Furthermore, another study demonstrated that the PLR significantly decreased in patients with prediabetes and in the early stages of diabetes (35). In the present study, the GLR was assessed in lieu of the NLR because the neutrophil count was not measured specifically; nevertheless, the GLR can represent the NLR since neutrophils account for the majority of granulocytes (23). Consequently, our results showed an increased GLR and a decreased PLR in the MHW group, which seemed to have a high risk of type 2 diabetes; these results are in line with those of previous studies. In other words, the GLR and PLR results in this study might be associated with the risk of type 2 diabetes, as demonstrated by the glucose-related markers (insulin, HOMA-IR, and adiponectin) measured in the present study.

FA profiles have been reported to reflect inflammatory status and to be able to cause chronic conditions (10, 39–41). Ferrucci et al. (39) revealed that total ω-3 PUFAs showed negative correlations with proinflammatory markers, such as IL-6, TNF-α, and CRP, whereas they showed positive correlations with anti-inflammatory markers, such as soluble IL-6 receptor, IL-10, and transforming growth factor-β. Another study also demonstrated that the plasma FA composition seemed to be linked to the inflammation statuses of overweight subjects based on increased IL-6 and CRP levels (41). Last, compared to healthy subjects, a higher arachidonic acid (ω-6 PUFA) level was observed in the plasma phospholipids of patients with type 2 diabetes (42). The present study also showed that the WBC count, a comprehensive indicator of inflammatory status, interacted with ω-3 PUFAs and dihomo-γ-linolenic acid as independent predictors of WBC level changes based on the multivariate linear regression analysis results. Thus, although the exact mechanism was not fully elucidated, we suggest that plasma FAs, especially total ω-3 PUFAs and dihomo-γ-linolenic acid, might be linked to WBC level-related type 2 diabetes risk.

**Figure 4:**
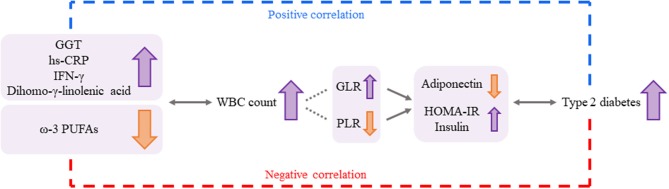
A possible association among the independent predictors for changes in WBC levels, the WBC count, and the risk for type 2 diabetes. GGT, hs-CRP, IFG-γ, ω-3 PUFAs, and dihomo-γ-linolenic acid could be considered independent predictors for changes in WBC levels, and the altered WBC levels were associated with the risk of type 2 diabetes by having relationships with glucose-related markers (adiponectin, HOMA-IR, and insulin). Changes in GLR and PLR according to WBC levels can underpin these associations more strongly. Thus, the independent predictors (inflammatory markers and plasma FAs) may have a potential role in increasing the risk of type 2 diabetes by elevating WBC levels. GGT: γ-glutamyltransferase. GLR, granulocyte-to-lymphocyte ratio; HOMA-IR, homeostatic model assessment of insulin resistance; hs-CRP, high-sensitivity C-reactive protein; IFN, interferon; PLR, platelet-to-lymphocyte ratio; PUFAs, polyunsaturated fatty acids; WBC, white blood cell.

This study has limitations. First, the study was conducted with a small sample size, and the results are hard to generalize for other populations. Second, we attempted to keep the stringent criteria to avoid potential confounder, but study subjects' socioeconomic status, which has been reported to be associated with WBC count change (43), was not considered as a confounding factor. Third, the underlying mechanisms of how the independent predictors affect WBC level alteration could not be thoroughly explained by our data, and only previous studies could support the results of this study. Nonetheless, taken together, these results suggest that GGT, hs-CRP, IFN-γ, ω-3 PUFAs, and dihomo-γ-linolenic acid can be considered independent predictors for changes in WBC levels and that altered WBC levels are associated with the risk of type 2 diabetes by having relationships with glucose-related markers, including insulin, HOMA-IR, and adiponectin (Figure 4). Therefore, we suggest that these inflammatory markers and plasma FAs may have a potential role and clinical relevance in increasing type 2 diabetes risk by elevating WBC levels in individuals without any chronic diseases but with borderline abnormal WBC levels. Finally, checking these biomarkers along with WBC levels can be helpful to prevent the chronic condition, especially the risk of type 2 diabetes.

## Data Availability Statement

The datasets generated for this study are available on request to the corresponding author.

## Ethics Statement

The study participants were fully given study explanation and provided written consent. The Institutional Review Board of Yonsei University reviewed and approved the study, which complied with the principles in the Declaration of Helsinki.

## Author Contributions

GS conducted the experimental analyses, interpreted the data, drew up the manuscript draft, and arranged the manuscript. KJ conducted the experimental analyses. MK designed the study and provided funding. JL provided the experimental setup and samples. HY designed the study, conducted the statistical analyses, interpreted the data, arranged the manuscript, and provided funding. All authors carefully reviewed the final manuscript and approved it for publication.

### Conflict of Interest

The authors declare that the research was conducted in the absence of any commercial or financial relationships that could be construed as a potential conflict of interest.
